# Impact of season on the association between vitamin D levels at diagnosis and one-year remission in early Rheumatoid Arthritis

**DOI:** 10.1038/s41598-020-64284-x

**Published:** 2020-04-30

**Authors:** M. Herly, K. Stengaard-Pedersen, P. Vestergaard, R. Christensen, S. Möller, M. Østergaard, P. Junker, M. L. Hetland, K. Hørslev-Petersen, T. Ellingsen

**Affiliations:** 1 Research Unit of Rheumatology, Department of Clinical Research, University of Southern Denmark, Odense University Hospital, Odense, Denmark; 2 Department of Rheumatology, Diagnostic Center, Silkeborg, Denmark; 3 Odense Patient data Explorative Network (OPEN), Odense University Hospital and Department of Clinical Research, University of Southern Denmark, Odense, Denmark; 4 Department of Rheumatology, Aarhus University Hospital and Institute of Clinical Medicine, Aarhus University, Aarhus C, Denmark; 5 Department of Clinical Medicine and Endocrinology, Steno Diabetes Center North Jutland, Aalborg University Hospital, Aalborg, Denmark; 6 Musculoskeletal Statistics Unit, The Parker Institute, Bispebjerg and Frederiksberg Hospital, Copenhagen, Denmark; 7 Center for Rheumatology and Spine Diseases and DANBIO, Centre for Head and Orthopaedics, Rigshospitalet, Glostrup, Denmark; Department of Clinical Medicine, University of Copenhagen, Copenhagen, Denmark; 8 Department of Rheumatology, King Christian 10th Hospital for Rheumatic Diseases, South Jutland Hospital, Institute of Regional Health Services, University of Southern Denmark, Sønderborg, Denmark

**Keywords:** Prognostic markers, Rheumatoid arthritis

## Abstract

The study evaluates associations between serum vitamin D metabolites at diagnosis and one-year remission, in early diagnosed rheumatoid arthritis(RA). The CIMESTRA-cohort comprised 160 newly diagnosed RA patients, treated aiming at remission. Vitamin D supplementation was recommended according to national guidelines. D_total_(25OHD_2_ + 25OHD_3_) was dichotomized at 50 nmol/L, 1,25(OH)_2_D was categorized in tertiles. Primary outcome was remission(DAS28-CRP ≤ 2.6) after one year. Associations were evaluated using logistic regression, further adjusted for pre-specified potential confounders: Age, sex, symptom-duration before diagnosis, DAS28-CRP and season of diagnosis. Results are presented as Odds Ratios(OR) with 95% Confidence Intervals(95%CIs). In univariate analyses, neither D_total_ nor 1,25(OH)_2_D were associated with remission. In adjusted analyses, low D_total_ was associated with higher odds for remission; OR 2.6, 95%CI (1.1; 5.9) p = 0.03, with season impacting results the most. One-year remission was lower in patients with diagnosis established at winter. In conclusion, low D_total_ at diagnosis was associated with increased probability of achieving one-year remission in early RA when adjusting for covariates. Diagnosis in winter was associated with lower odds for one-year remission. Results suggest that season act as a contextual factor potentially confounding associations between vitamin D and RA disease-course. The finding of low D_total_ being associated with higher one-year remission remains speculative.

## Introduction

Mounting evidence suggests vitamin D as an important immune-modulator^1,2^. Circulating 25OHD_3_ is produced in the skin upon sun-exposure, dependent on e.g. season and latitude^3^, and circannual variation in 25OHD is well-known in Northern latitude^4^. 25OHD serves as substrate for conversion into the active metabolite; 1,25(OH)_2_D, both in the kidneys^3^ and locally in macrophages, dendritic cells and lymphocytes^5^. Immunomodulation by sufficient levels of 1,25(OH)_2_D includes altered differentiation and maturation of dendritic cells, macrophages and lymphocytes^5^, and inhibition of pro-inflammatory cytokines^6^. The main-effect of vitamin D on the immune system is to balance the immune-response away from the self-aggressive Th1/Th17-response and towards the tolerogenic Th2/Th-regulatory response^7,8^.

In Rheumatoid Arthritis (RA), a chronic inflammatory symmetric arthritis^9^, low levels of 25OHD are reported to be associated with markers of disease activity in cross-sectional settings^10,11^. 1,25(OH)_2_D is rarely evaluated, but seems to be inversely associated with several markers of disease activity in cross-sectional settings^12^.

An aggressive aiming-at-remission treatment strategy is the golden standard in RA^13^, and the immune-modulating activity of vitamin D and association with disease activity in cross-sectional studies suggests potential association between vitamin D and remission in RA. To the best of knowledge, only two rather small longitudinal studies evaluate associations between serum 25OHD at time of RA diagnosis and remission during follow-up^14,15^. Both studies report lower odds of remission in RA-patients with low 25OHD at diagnosis. Association between 1,25(OH)_2_D and remission was not evaluated.

The objectives of the current study were, in 158 early diagnosed, treatment-naïve Danish RA patients, to measure serum D_total_ and 1,25(OH)_2_D at time of diagnosis, and to analyze for associations with remission evaluated as Disease Activity Score based on 28 joints and C-reactive Protein (DAS28-CRP) ≤ 2.6 after one year. This was done to test the pre-specified hypothesis that baseline vitamin D metabolites are associated with achieving remission in early RA.

## Results

At diagnosis, median D_total_ was 53.7 nmol/l, IQR 36-67. Median 1,25(OH)_2_D was 95.5 pmol/l, IQR 70–119. Sixty-seven patients (42%) had D_total_ below 50 nmol/L.

**Figure 1:**
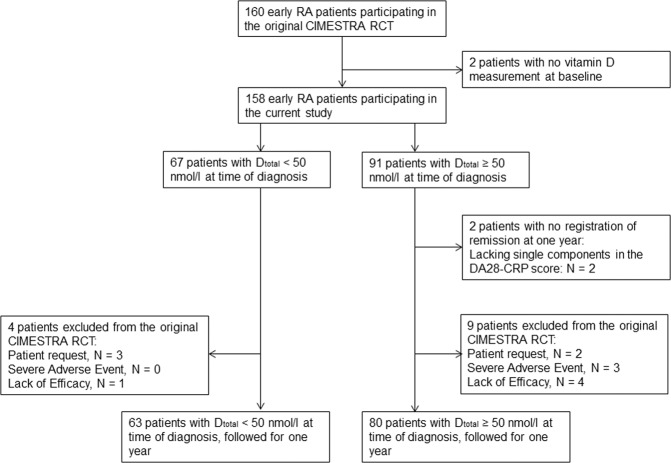
Patient flow-diagram for year one in the original CIMESTRA study, according to D_total_ status at diagnosis - Abbreviations: D_total_: the sum of 25OHD_2_ and 25OHD_3_.

Obesity (BMI > 30 kg/m^2^) was significantly more prevalent in the low D_total_ group (30% versus 13%, p = 0.01) as a result of weight being higher (median 79 kg versus 73 kg, p = 0.03) and 1,25(OH)_2_D was significantly lower (median 82 pmol/L versus 110 pmol/L, p < 0.001) No other baseline variables were significantly different between D_total_ groups. (See Table 1**)** One-hundred-and-forty-three of the 158 patients included at baseline had disease activity evaluated one year after diagnosis, and were eligible for evaluation of remission. (See Fig. 1).

**Table 1:** Baseline characteristic according to D_total_ status at diagnosis.

	Low D_total_ (<50 nmol/L) N = 67 (42%)	Normal D_total_ (≥50 nmol/L) N = 91 (58%)	Group comparison (p-value)	Total N = 158
Age at inclusion, Years	Median 52 IQR 44–63	Median 53 IQR 41–63	0.83	Median 53 IQR 43–63
Female sex	N = 42 (63%)	N = 62 (68%)	0.48	N = 104 (66%)
Symptom duration prior to diagnosis, months	Median 3.3 IQR 2.4–4.8	Median 3.2 IQR 2.5–4.4	0.81	Median 3.2 IQR 2.5–4.6
Ciclosporin (yes)	N = 36 (54%)	N = 42 (46%)	0.35	N = 78 (49%)
ACPA-status positive	N = 39 (58%)	N = 53 (58%)	0.99	N = 92 (58%)
IgM-RF-status positive	N = 39 (58%)	N = 63 (69%)	0.15	N = 102 (65%)
NTJ (0 to 28)	Median 10 IQR 6–16	Median 10 IQR 5–15	0.25	Median 10 IQR 5–15
NSJ (0 to 28)	Median 9 IQR 5–12	Median 8 IQR 5–12	0.67	Median 8 IQR 5–12
VAS_patient-global_ (0 to 100 mm)	Median 50 IQR 26–72	Median 50 IQR 29–72	0.61	Median 50 IQR 29–72
VAS_patient-pain_ (0 to 100 mm)	Median 48 IQR 27–72	Median 46 IQR 29–72	0.96	Median 48 IQR 29–70
VAS_doctor-global_(0 to 100 mm)	Median 58 IQR 42–70	Median 55 IQR 36–68	0.41	Median 58 IQR 38–68
ESR, mm/hour	Median 32 IQR 9–48	Median 23 IQR 11–47	0.65	Median 28 IQR 10–47
CRP, nmol/L	Median 24 IQR 9–51	Median 17.3 IQR 9–39	0.19	Median 20.2 IQR 9–43
DAS28-CRP (0 to 9)	Median 5.3 IQR 5.6–6.1	Median 5.2 IQR 4.4–5.9	0.32	Median 5.3 IQR 4.5–5.9
HAQ (0 to 3)	Median 1 IQR 0.5–1.5	Median 0.9 IQR 0.4–1.5	0.88	Median 0.7 IQR 0.4–1.5
Creatinine µmol/L	Median 75 IQR 67–82	Median 77 IQR 68–83	0.53	Median 76 IQR 67–82
**BMI** ≥ **30 (kg/m**^**2**^**)**	**N** = **20 (30%)**	**N** = **12 (13%)**	**0.01**	**N** = **32 (20%)**
**Weight, kg**	**Median 79** **IQR 64–92**	**Median 73** **IQR 64–82**	**0.03**	**Median 76** **IQR 64–85**
Height, cm	Median 170 IQR 166–175	Median 170 IQR 164–175	0.76	Median 170 IQR 165–175
**1,25(OH)**_**2**_**D, pmol/L**	**Median 82** **IQR 65–99**	**Median 110** **IQR 80–139**	**<0.001**	**Median 95.5** **IQR 70–119**
**Season of diagnosis (Winter)**	**N** = **44 (66%)**	**N** = **37 (41%)**	**0.002**	**N** = **81 (51%)**

Abbreviations: D_total_: The sum of 25OHD_2_ and 25OHD_3_. ACPA: Anti Citrullinated Protein Antibodies, IgM-RF: Immunoglobulin M Rheumafactor, NTJ: Number of Tender Joints, NSJ: Number of Swollen Joints, VAS: Visual Analogue Score, ESR: Erythrocyte Sedimentation rate, CRP: C-reactive protein, DAS28-CRP Disease Activity Score, based on 28 joint count and CRP. HAQ: Health Assessment Questionnaire, BMI: Body Mass Index. Data as observed. Significant results in bold.

### Results of univariate logistic regression

Diagnosis established at winter compared to summer (OR 0.4, 95% CI (0.2; 0.9) p = 0.02), Number of Tender Joints (NTJ) (OR 0.9; 95% CI (0.88; 0.98) p = 0.01) and DAS28-CRP (OR 0.7, 95% CI (0.5; 0.9) p = 0.01) at time of diagnosis were significantly inversely associated with one-year remission, independently of vitamin D metabolite levels. This indicates that it is less likely that patients diagnosed during the winter season and in patients with high NTJ or DAS28-CRP at time of diagnosis, will achieve remission. [See appendix Table 1].

### Primary analysis, evaluating D_total_ groups

At year one, 96 (61%) of the 143 patients were in remission, 45 (71%) from the group with low D_total_ at diagnosis, and 51 (64%) from the group with normal D_total_ at diagnosis. (See Table 2).

**Table 2:** Achieving remission according to D_total_ at diagnosis.

	Low D_total_ (<50 nmol/L)	Normal D_total_ (≥50 nmol/L)	Contrast between groups
Primary outcome: Remission. (%)	N = 45 (71%)	N = 51 (64%)	Crude analysis^a^: OR 1.4, 95% CI (0.7; 2.7) p = 0.33 Logistic regression^b^: OR 1.4, 95% CI (0.7; 2.9) p = 0.34 Adjusted logistic regression^c^: **OR 2.6, 95% CI (1.1; 5.9) p** = **0.03**
Best-case scenario assuming the 15 patients not evaluated for year on, all did achieved remission	N = 49 (73%)	N = 62 (68%)	Crude analysis^a^: OR 1.3, 95% CI (0.6; 2.6) p = 0.50 Logistic regression^b^: OR 1.3, 95% CI (0.6; 2.6) p = 0.51 Adjusted logistic regression^c^: OR 2.0 95% CI (0.9, 4.3) p = 0.09
Worst-case scenario assuming the 15 patients not evaluated for year one, all did not achieved remission	N = 45 (67%)	N = 51 (56%)	Crude analysis^a^: OR 1.6, 95% CI (0.8; 3.1) p = 0.16 Logistic regression^b^: OR 1.6, 95% CI (0.8; 3) p = 0.2 Adjusted logistic regression^c^: OR 2.9, 95% CI (1.3; 6.2) p = 0.01
Change in disease activity markers after 1 year according to D_total_ group at time of diagnosis			
Change in HAQ	Mean: −0.6 95% CI (−0.7; −0.5)	Mean: −0.6 95% CI (−0.7; −0.5)	Least Squares Mean Difference: Crude analysis: −0.1, 95% CI (−0.2;0.1) p = 0.48
Change in NTJ	Mean: −9.7 95%CI (−10.9; 8.8)	Mean: −8.6 95% CI (−9.5; 7.8)	Least Squares Mean Difference: Crude analysis: 1.1, 95% CI (−0.2; 2.4) p = 0.09
Change in NSJ	Mean: −8.8 95% CI (−9.3; −8.3)	Mean: −8.1 95% CI (−8.1; −7.7)	Least Squares Mean Difference: Crude analysis: 0.7, 95% CI (0.0; 1.3) p = 0.04
Change in VAS_doctor global_	Mean: −47.7 95% CI (−51; −44.4)	Mean: −44.7 95% CI (−47.5; −41.8)	Least Squares Mean Difference: Crude analysis: 3.2, 95% CI (−1.3; 7.5) p = 0.17
Change in VAS_patient-pain_	Mean: −29.7 95% CI (−34.9; −24.5)	Mean: −28.2 95% CI (−32.7; −23.6)	Least Squares Mean Difference: Crude analysis: 1.6, 95% CI (−5.3; 8.5) p = 0.65
Change in VAS_patient global_	Mean: −31.6 95% CI (−37; −26.2)	Mean: −28.9 95% CI (−33.7; −24.1)	Least Squares Mean Difference: Crude analysis: 2.7, 95% CI (−4.5; 10) p = 0.46
Change in ESR, mm/hour	Mean: −12.8 95% CI (−16.3; −9.3)	Mean: −14.2 95% CI (−17.3; −11.1)	Least Squares Mean Difference: Crude analysis: −1.4, 95% CI (−6.2; 3.3) p = 0.55
Change in CRP, mg/l	Mean: −21.1 95% CI (−25.2; −16.9)	Mean: −20.4 95% CI (−24.1; −16.7)	Least Squares Mean Difference: Crude analysis: 0.6, 95% CI (−4.9; 6.2) p = 0.82
Change in DAS28-CRP	Mean: −3 95% CI (−3.3; −2.7)	Mean: −2.7 95% CI (−2.9; −2.4)	Least Squares Mean Difference: Crude analysis:0.3, 95% CI (−0.1; 0.7) p = 0.08

^a^Crude logistic regression; D_total_ group as independent variable, no other covariates.

^b^Simple logistic regression, further containing age at diagnosis and sex.

^c^Multiple logistic regression, further adjusted for symptom-duration prior to diagnosis, DAS28-CRP at diagnosis and diagnosis established in winter (November to April).

For all comparisons, the group having normal D_total_ (i.e defined as “not low”) is the reference.

Abbreviations: D_total_: The sum of 25OHD_2_ and 25OHD_3_. BMI: Body Mass Index, ACPA: Anti Citrullinated Protein Antibodies, IgM-RF: Immunoglobulin M Rheumafactor, NTJ: Number of Tender Joints, NSJ: Number of Swollen Joints, VAS: Visual Analogue Score, HAQ: Health Assessment Questionnaire, CRP: C-reactive protein, DAS28-CRP Disease Activity Score, based on 28 joint count and CRP.

No significant association was found between baseline D_total_ group and remission rates in the crude model; OR 1.4, 95% CI (0.7; 2.7) p = 0.33, whereas the fully adjusted model showed statistical significantly increased odds for one year remission; OR 2.6, 95% CI (1.1; 5.9) p = 0.03, in patients with low D_total_ at time of diagnosis compared to patients with normal D_total_. (See Table 2) For details on the definition of crude and adjusted models, see the method section. Propensity score analysis including several other factors, potentially affecting the results, did not significantly alters the estimate; OR 2.5, 95% CI (1.1; 5.8) p = 0.03, indicating that the further included factors have only negligible influence. In the fully adjusted model, remission was further inversely associated with diagnosis established in winter; OR 0.3 95% CI (0.1; 0.7) p < 0.01) and DAS28-CRP at diagnosis; OR 0.6 95% CI (0.4; 0.9) p = 0.01 and directly associated with age at diagnosis; OR 1.04, 95% CI (1.0; 1.1) p = 0.03. Stepwise inclusion of the pre-specified variables sex, age, symptom duration, DAS28-CRP at diagnosis and season at diagnosis indicates that especially adjustment for season impacts the estimates in the fully adjusted model. (See Table 3). There was no significant interaction between D_total_ levels and season of diagnosis (p = 0.7).

**Table 3:** Stepwise regression, including a priori chosen variables one by one.

	OR for remission with inclusion of the mentioned variables
Crude analysis (D_total_ and remission)	OR 1.4, 95% CI (0.7;2.9) p = 0.33
Adjusted for sex	OR 1.4, 95% CI (0.7;2.9) p = 0.33
Adjusted for sex and age	OR 1.4, 95% CI (0.7; 2.9) p = 0.33
Adjusted for sex, age and disease-duration	OR 1.4, 95% CI (0.7;3.01) p = 0.30
Adjusted for sex, age, disease-duration and DAS28	OR 1.8, 95% CI (0.8;2.9) p = 0.13
Adjusted for sex, age, disease-duration, DAS28 and diagnosis established at winter.	OR 2.6, 95% CI (1.1; 5.9) p = 0.03

Abbreviations: D_total_: The sum of 25OHD_2_ and 25OHD_3_. DAS28-CRP Disease Activity Score, based on 28 joint count and CRP.

Model fit was tested to evaluate which model best describes the association between vitamin D metabolites, one year remission and the chosen, potential confounding variables: A likelihood-ratio test showed that fully adjusting the model improved the model fit significantly, whereas the partially adjusted model (including only sex and age at inclusion) did not improved the model fit compared to the crude model. In addition, Akaike Information criterion (AIC) decreased and Bayesian Information Criterion (BIC) increased when including all confounding variables. As we put emphasis on AIC, this is interpreted as the fully adjusted model being the model which most adequately describes the real world, resulting in the best balance between number of included variables and precision.

To estimate whether missing data for one-year remission affects the results, best- and worse case-scenarios were evaluated. These suggested potentially consequences of missing data, as estimates became insignificant in best-case scenarios. (See Table 2).

### Secondary analysis, evaluating 1,25(OH)_2_D tertiles

Associations between the active vitamin D metabolite and one-year remission were evaluated similar to the primary analyses for D_total_. No difference existed in odds of remission between patients with the lowest 1,25(OH)_2_D tertile compared to highest, neither in the univariate model; OR 1.3, 95% CI (0.5; 3.2) p = 0.59, nor in the fully adjusted model; OR 1.8, 95% CI (0.6; 4.9) p = 0.28.

Remission was negatively associated with diagnosis established at winter; OR 0.4 95% CI (0.2; 0.9) p = 0.03, and DAS28-CRP at diagnosis; OR 0.6 95% CI (0.4; 0.9) p = 0.01. [See appendix Table 2].Applying likelihood-ratio-test, both the partially and fully adjusted model showed significantly better fit than the crude model. AIC decreased with each successive adjustment, while BIC decreased from crude to fully adjusted model.

## Discussion

Main finding is that the inverse association between serum D_total_ status at diagnosis and one year remission exists only in the model adjusted for season. Although largely unexplained, being diagnosed with RA in the winter is independently associated with lower odds for remission after one year of intensive treatment, suggesting that season confounds the association between vitamin D and RA disease activity.

The immunomodulatory properties of vitamin D are exerted through local conversion of 25OHD into 1,25(OH)_2_D^1,5^. Subjects with low serum D_total_ lack substrate for the local production of 1,25(OH)_2_D^16,17^, increasing the risk of an auto-aggressive Th1/Th17 response^18^. We surmise that our results support the potential existence of a “low vitamin D RA-subtype”, which upon anti-inflammatory treatment and relevant vitamin D supplementation is easily treated to remission. On the contrary, subjects with normal D_total_ levels and thus sufficient substrate for local 1,25(OH)_2_D production, who develop RA may be representative of an immune system being more imbalanced and distressed, and thus less susceptible to achieve remission, despite relevant treatment. Alternatively, our results indicate the presence of a “Winter-diagnosed RA-subtype”. Patients diagnosed at winter will inevitably have one-year remission evaluated the following winter, where the seasonal impact on disease activity will decrease probability of remission. Though, our study-design does not allow us to speculate whether “winter-diagnosed RA” is less prone to achieve remission in the summer, as we have no half-yearly evaluations.

Our results conflict with other studies evaluating vitamin D and remission in early RA: As opposed to the current study, the comparable studies did not adjusted for season in the statistical analyses, but handled potential annual impact through study design: One study is located in Columbia, a tropical country with no seasons^15^, while the Italian study^14^ only evaluated serum 25OHD in the autumn-winter-period, to minimize the seasonal impact. However, RA is diagnosed all year round, and circannual changes in light and darkness is unavoidable in Scandinavia. We find the current study to be representative of a Scandinavian, early diagnosed RA-cohort, and further sheds light on an important contextual factor in evaluation of disease activity in early RA.

The impact of season on vitamin D levels is well-known^3,4^ Similarly, season impacts the incidence and course of RA^19–21^, likely owing to circadian rhythms affecting pro-inflammatory cytokines^22,23^. This may be explained by effects of seasonal changes in vitamin D affecting the immune system^24^, perhaps through seasonal impact on relevant genes, independent of vitamin D metabolism^25,26^.

Our study design implies that one year remission is evaluated the same season as diagnosis was established, and the impact of season on disease activity at diagnosis will be mirrored in the remission-criteria applied one year after. Thus, the probability to observe one-year remission will be lower in patients diagnosed in winter. As D_total_ levels are lower in winter, we suggest this being a plausible explanation to our main finding: season is a strong confounder in the relationship between vitamin D and disease activity in RA. As season independently predicted one-year remission, we further suggest our results to be indicative of season of diagnosis being an important contextual factor in evaluation of RA disease-course. We advocate future research to take season into account when evaluating disease activity in early RA.

The primary outcome; remission, is evaluated as DAS28; a composite of subjective and objective disease-activity markers weighting pain and inflammation. Pain may persist in DAS28-remission^27^, whereas 1,25(OH)_2_D supplementation is reported to significantly relieve pain in early RA^28^. As the CIMESTRA strategy included increased awareness of vitamin D intake, we assume that an eventual painful proximal myopathy owing to low vitamin D was improved during follow-up. In RA, 25OHD levels may be correlated to dietary intake^29^, while improvement of low serum vitamin D upon supplementation in RA is associated with improved treatment response^30^. Moreover, RA patients with low 25OHD and no vitamin D supplementation are less frequently in remission, compared to patients receiving relevant supplementation^31^. Likewise, abstaining from correction of low vitamin D levels is associated with increased recurrence-rates in some^32^, but not all studies^33^. However, the current study is not an interventional study investigating effects of vitamin D supplementation on remission. Patients were supplemented after individual physicians´ evaluation of vitamin D and calcium intake, with no knowledge of actual serum D_total_ level. Suggesting that sufficient vitamin D intake, concomitant to aggressive anti-inflammatory treatment, might partly explain the results, is only hypothesis-generating, despite plausible underlying biological associations supporting the hypotheses^1^. Though, data concerning which patients received supplementation is not available.

If a causal association between low vitamin D levels and high RA disease activity exists, it seems reasonable that patients with more active disease shows better treatment response upon the aggressive treatment-regimen and relevant vitamin D intake, thus explaining the higher remission-rate in the group with lower D_total_. Furthermore, if a “low vitamin D RA subtype” exists, and low vitamin D levels are associated with higher disease activity and pain^11^, such patients may receive higher doses of anti-inflammatory medication, and be more likely to achieve remission, partly explaining the results.

Limitations of the current study include restrictions owing to the use of post-hoc analyses in a parental RCT, designed for other purposes: The study population is rather small, supported by several estimates having large 95% CIs. However, to the best of knowledge, our study almost doubles observations upon which estimates can be made, compared to other studies evaluating vitamin D and remission in early RA^14,15^. The parental CIMESTRA RCT investigated the additive effect of ciclosporine or placebo to an aggressive, treat-to-target strategy, and 50% received ciclosporine. Vitamin D metabolites evaluated in the current study were obtained prior to treatment, and were not affected by ciclosporine treatment. No adjustment for ciclosporine is done, as no significant association was found between ciclosporine and one year remission^34^.

Possible unmeasured residual-confounders must be considered: Relevant markers of calcium metabolism, such as calcium, phosphorous and parathyroid hormone are not evaluated. Neither, information concerning sun-exposure, the main-cause to cutaneous vitamin D production, is assessed. Besides, vitamin D levels have been reported to vary according to meat consumption^35–37^. However, we are unable to account for meat-consumption in the current study.

Another potential limitation is the dichotomization of D_total,_ applied in the current study: Cutpoint defining sufficient vitamin D levels varies greatly, depending on e.g. age, race, weight, sun-exposure and diet^3^. The internationally accepted cut-point defining low vitamin D is 50 nmol/l^38^. We acknowledge that dichotomization might lead to loss of more subtle details, and that it has been argued that higher levels of vitamin D might be necessary for better health outcomes^39^. However, we find that the use of a well-known cut-point, and the comparability to the few similar studies increase the external validity, interpretation and clinical use of our results.

To avoid overfitting our statistical models, we evaluated model fits by AIC and concluded that the fully adjusted model was the most realistic model. As different modelling scenarios resulted in inconsistency among results, we advocate for reproduction of our results before firm conclusions are made

We believe that our data can be applied to a Scandinavian population with newly diagnosed RA. Despite data collection at the millennium, the aggressive treat-to-target strategy is still the recommendations nowadays, increasing the external validity. Our results need to be reproduced, preferably in sufficiently powered studies with vitamin D supplementation guided by serum vitamin D measurements, alongside to aggressive treatment and adjustment for season.

In conclusion, low vitamin D_total_ at time of diagnosis in early, treatment-naïve RA is associated with increased odds of achieving remission after one year, when adjusting for relevant confounding variables. Conceivably, this finding is mainly attributable to seasonal variation *per se* with low vitamin D levels as a potential amplifier. In order to elucidate the relationship between vitamin D status, disease activity and season of the year, follow-up visits at 6 months interval may be useful. In the meantime, clinicians and researchers should be aware of the potential impact by season on RA disease presentation.

## Methods

### Patients and outcomes

One-hundred-and-sixty patients were recruited from five Danish University Clinics from October 1999 to October 2002 in the CIMESTRA-study. Eligibility criteria and treatment-strategy are described elsewhere^34^. One-hundred-and-fifty-eight patients had D_total_ (the sum of 25OHD_2_ and 25OHD_3_) and 1,25(OH)_2_D) measured prior to inclusion and treatment. One-Hundred-and-forty-three patients had DAS28-CRP calculated after one year, and were included in the current study. Patients were allocated to D_total_ group at time of diagnosis, dichotomized as low: D_total_ < 50 nmol/L or normal: D_total_ ≥ 50 nmol/L. Patients who reported insufficient vitamin D and calcium intake at time of diagnosis received daily oral supplements of vitamin D_3_ (800 IE/10 mcg) and calcium (1500 mg), according to national guidelines. Treating physician decided eventual need for supplementation unaware of actual serum vitamin D levels, as serum levels were evaluated from biobank for this current study. NTJ, Number of Swollen Joints – 28 joint count (NSJ), Visual Analogue Scores (VAS): VAS_global-doctor_, VAS_global-patient_, VAS_pain-patient_, CRP, DAS28-CRP, Health Assessment Questionnaire (HAQ), Anti Citrullinated Protein Antibodies (ACPA), Immunoglobulin M-Rheuma Factor (IgM-RF) and season of diagnosis (Winter defined as diagnosis established between November and April) were evaluated at time of diagnosis. DAS28-CRP ≤ 2.6 at one year was defined as remission. (See Fig. 1).

### Measurement of the vitamin D metabolites

25OHD_2_ and 25OHD_3_ in serum were analyzed with isotope dilution liquid chromatography-mass spectrometry (LC-MS/MS) using calibrators traceable to international standard reference material NIST SRM 972^40^. Mean coefficients of variation (CVs) of 25OHD_3_ were 9.6% and 8.1% at 25 nmol/L and 48 nmol/L, respectively, and for 25OHD_2_, 8.5% and 8.0% at levels of 23 and 64 nmol/L, respectively^41^.

1,25(OH)_2_D was analyzed by radio Immuno Assays (RIA)^42^ after immune-extraction of the samples. (1,25-dihydroxy vitamin D RIA, IDS, Boldon, UK) Mean intra-assay (well-to-well) CV were 6.8% and 9.0% at levels of 90 and 220 pmol/L, respectively.

### Routine laboratory assessments

IgM-RF was detected by enzyme-linked immunosorbent assay (ELISA). ACPA-IgG antibodies were determined by a second-generation ELISA (Immunoscan RA kit, Euro-diagnostica AB, Malmo, Sweden) Serum CRP was measured using standard laboratory measures.

### Statistical methods

Differences between D_total_ groups for dichotomous variables at time of diagnosis were evaluated by Chi^2^ test. Wilcoxon-Rank-Sum test evaluated differences in continuously variables. Underlying assumptions were evaluated through study-design.

Univariate, logistic regression was performed for D_total_, comparing levels <50 nmol/L to levels ≥50 nmol/L, in predicting DAS28-CRP ≤ 2.6 after one year. This is defined as the “crude model”. In the model defined as the “partially adjusted model”, sex and age at diagnosis were included, whereas symptom-duration prior to diagnosis, DAS28-CRP and diagnosis established at winter were further included in the fully adjusted model. These variables were chosen prior to analysis because of their previously reported ability to predict clinical remission^43^. or close physiological relation to vitamin D^4^. Despite patients originally randomized 1:1 to ciclosporine, it was decided not to adjust for ciclosporine, as no association with one-year remission was found for ciclosporine in the original CIMESTRA study^34^.

Further aiming at evaluating the delicate interplay between variables included in the model, a step-by-step approach was used, including sex, age, symptom duration, DAS28-CRP at diagnosis and season of diagnosis in a logistic regression one by one.

The model was subsequently evaluated for interaction between vitamin D and season of diagnosis, as a close relationship exists between season and circulating D_total_. Likelihood ratios tests were used to evaluate model fit. AIC and BIC assesses the model fit when taking number of co-variates included into account, and thus estimates the risk of over-fitting. Emphasis is put on AIC, while BIC is also presented.

To test the robustness of the model, propensity score analysis was applied to evaluate the potential biasing impact of other variables on the outcome. The propensity score analysis included BMI, ACPA, IgM-RF, NSJ, NTJ, VAS_global-patient_, HAQ and CRP at time of diagnosis to determine, if confounding by any of these factors was biasing the results.

The potential impact of the missing remission-data of the 15 patients not included in year one was evaluated using best- and worst case scenarios, coding all missing patients as being either in remission (“Best case”) or not in remission (“Worst case”).

Concerning 1,25(OH)_2_D, similar analyses were performed as for D_total_, comparing lowest and middle tertile to highest tertile.

Significance is defined as the 5% level. Stata IC 15^44^ is used for statistical analyses.

## Ethics and Consent

The study complies with the Oviedo and Helsinki declarations. The protocol was approved by Ethics Committee Region Midt (M-1959–98) and registered at www.clinicaltrials.gov (NCT00209859). All patients received verbal and written information prior to enrolment, and gave written consent at baseline.

## Supplementary information

Appendix tables and appendix figure.
