# Supplementary material for: Impact of season on the association between vitamin D levels at diagnosis and one-year remission in early Rheumatoid Arthritis
Source: Sci Rep. 2020 Apr 30;10:7371. doi: 10.1038/s41598-020-64284-x (PMC7192905; doi:10.1038/s41598-020-64284-x)

Title page

Impact of season on the association between vitamin D levels at diagnosis and one-year remission in early Rheumatoid Arthritis.

M. Herly, K. Stengaard-Pedersen, P. Vestergaard, R. Christensen, S. Möller, M. Østergaard, P. Junker, M. L. Hetland, K. Hørslev-Petersen, T. Ellingsen.

Table legend: Appendix table 1: Associations between baseline variables and remission at one year.

Appendix table 1

Table legend: Associations between baseline variables and remission at one year.

Baseline variable:	Estimate (OR, 95% CI and p-value for difference)
D_{total} < 50 nmol/L	$OR_{Low\ vs\ Normal}$: 1.42, (95% CI 0.7; 2.9) p=0.33
1,25(OH)2D, tertiles, pmol/L	$OR_{low\ vs\ high}$ 1.28 (95% CI 0.5; 3.2) p=0.59
	$OR_{middle\ vs\ high}$ 0.60 (95% CI 0.3; 1.4) p=0.24
Male sex	OR 1.3, (95% CI 0.6; 2.8) p=0.77
Age (years)	OR 1.0, (95% CI 0.9; 1.0) p=0.49
Disease duration (weeks)	OR 0.82, (95% CI 0.6; 1.1) p=0.16
Adipositas (BMI > 30)	OR 0.7, (95% CI 0.3; 1.6) p=0.35
IgM-RF positivity	OR 1.1, (95% CI 0.6; 2.3) p=0.74
ACPA positivity	OR 1.2, (95%CI 0.61; 2.49) p=0.56
NSJ (0-28)	OR 1 (95% CI 0.9; 1.0) p=0.27
NTJ (0-28)	OR 0.9 (95% CI 0.9; 1) p= 0.01
$VAS_{global\text{-}patient}$ (0-100)	OR 1 (95% CI 0.9; 1.0) p=0.08
CRP, nmol/L	OR 1 (95% CI 0.9; 1.0) p=0.18
DAS28-CRP (0 to 9)	OR 0.7 (95% CI 0.5; 0.9) p=0.01
HAQ (0-3)	OR 0.7 (95% CI 0.5; 1.2) p=0.24
Diagnosis established at winter (November to April)	$OR_{Winter\ vs\ Summer}$ 0.4 (95% CI 0.2; 0.9) p=0.02
Abbreviations: D_{total}: The sum of $25OHD_2$ and $25OHD_3$, BMI: Body Mass Index, IgM-RF: Immunoglobulin M Rheumafactor, ACPA: Anti Citrullinated Protein Antibodies, NSJ: Number of Swollen Joints (28 joint count), NTJ: Number of Tender Joints (28 joint count) VAS;: Visual Analogue Score, HAQ: Health Assessment Questionaire	

Appendix table 2

Table legend: Achieving remission according to 1,25$(OH)_2$D at diagnosis

**Appendix table 2**	**1,25$(OH)_2$D lowest tertile**	**1,25$(OH)_2$D middle tertile**	**1,25$(OH)_2$D highest tertile**
Baseline Median and IQR	Median 65.5<br>IQR 57-71	Median 96.5<br>IQR 90–104	Median 138.5<br>IQR 123–175
In remission at one year. No. %	35 (65%)	29 (54%)	32 (64%)
Primary outcome: Remission. (%)	Crude analysis[a]<br>OR 1.3, 95% CI (0.5; 3.2)<br>p=0.59<br>Logistic regression[b]<br>OR 1.2, 95% CI (0.5; 3.1)<br>p=0.65<br>Logistic regression[c]<br>OR 1.8, 95% CI (0.6; 4.9)<br>p=0.28	Crude analysis[a]<br>OR 0.6 95% CI (0.3; 1.4)<br>p=0.24<br>Logistic regression[b]<br>OR 0.6, 95% CI (0.3; 1.4)<br>p=0.24<br>Logistic regression[c]<br>OR 0.7, 95% CI (0.3; 1.8)<br>p=0.47	Reference-group
Worst-case scenario	Crude analysis[a]:<br>OR 1.0, 95%CI (0.5; 2.3)<br>p=0.9<br>Logistic regression[b]:<br>OR 0.9, 95% CI (0.4; 2.1)<br>p=0.88 Adjusted logistic regression[c]:<br>OR 1.3, 95% CI (0.5; 3.2)<br>p=0.59	Crude analysis[a]:<br>OR 0.7: 95% CI (0.3; 1.4)<br>p=0.29<br>Logistic regression[b]:<br>OR 0.6; 95% CI (0.4; 1.4)<br>p=0.25<br>Adjusted logistic regression[c]:<br>OR 0.8, 95% CI (0.3; 1.8)<br>p=0.56	Reference-group
Best-case scenario	Crude analysis[a]:<br>OR 1.4, 95% CI (0.6; 3.3)<br>p=0.5<br>Logistic regression[b]:<br>OR 1.4, 95% CI (0.6; 3.4)<br>p=0.48<br>Adjusted logistic regression[c]:<br>OR 1.8, 95% CI (0.7;4.9)<br>p=0.23	Crude analysis[a]:<br>OR 0.6, 95% CI (0.3; 1.4)<br>p=0.24<br>Logistic regression[b]:<br>OR 0.6, 95% CI (0.3; 1.4)<br>p=0.25<br>Adjusted logistic regression[c]:<br>OR 0.7, 95% CI (0.3; 1.7)<br>p=0.43	Reference-group

[a]Crude logistic regression; D_{total} group as independent variable, no other covariates.
[b]Simple logistic regression, further containing age at diagnosis and sex
[c]Multiple logistic regression, further adjusted for symptom duration prior to diagnosis, DAS28-CRP at diagnosis and diagnosis established at winter (November to April)
For comparisons, the group having 1,25$(OH)_2$D in the highest tertile is the reference.

Appendix figure 1:

Figure legend: Worst and best case scenarios:

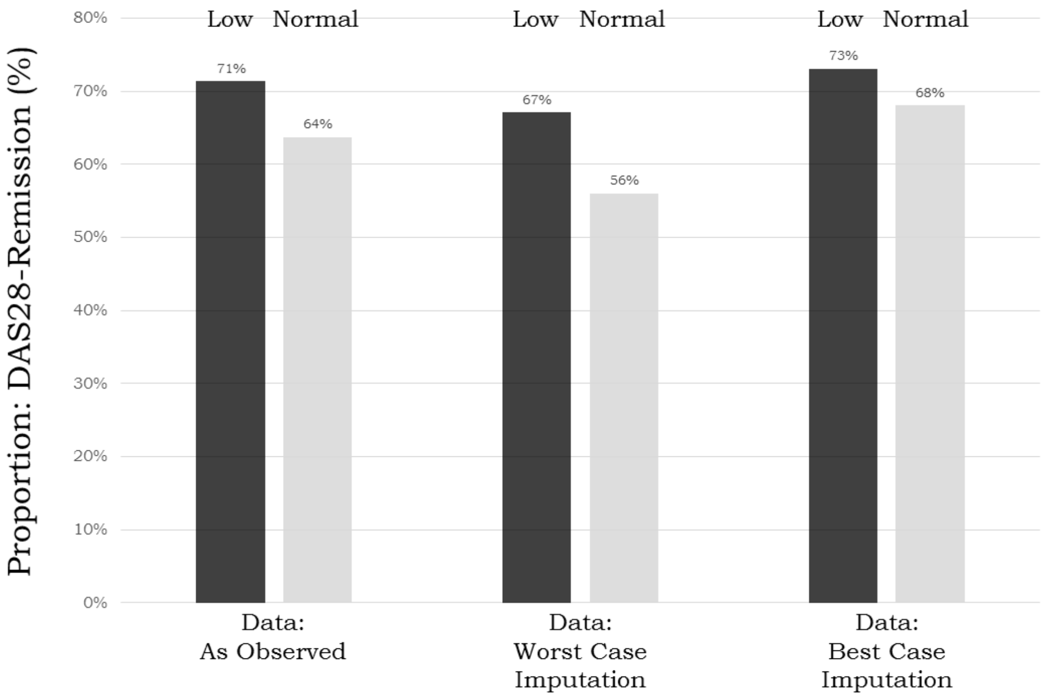

Supplement: Supplementary file 1 — Appendix tables and appendix figure. [file 41598_2020_64284_MOESM1_ESM.pdf]
